# Demographic characteristics and clinical profile of poor responders in IVF / ICSI: A comparative study

**DOI:** 10.4103/0974-1208.69343

**Published:** 2010

**Authors:** Nabaneeta Padhy, Shalu Gupta, Asmita Mahla, M Latha, Thangam Varma

**Affiliations:** Institute of Reproductive Medicine, Madras Medical Mission, Chennai – 600 037, India

**Keywords:** Gonadotrophin, ovarian response, pelvic surgery, poor responder

## Abstract

**BACKGROUND:**

Ovarian response varies considerably among individuals and depends on various factors. Poor response in IVF yields lesser oocytes and is associated with poorer pregnancy perspective. Cycle cancellation due to poor response is frustrating for both clinician and the patient. Studies have shown that women conceiving after poor ovarian response have more pregnancy complications like PIH and preeclampsia than women with normal ovarian response. In addition, poor ovarian response could be a predictor of early menopause. This paper studies various demographic and clinical profiles of poor responders and tries to look at the known and unknown factors which could contribute to poor ovarian response in IVF.

**MATERIALS AND METHODS:**

Data were collected retrospectively from 104 poor responders who had less than four oocytes at retrieval and compared with 324 good responders for factors like age, BMI, type of sub fertility, duration of sub fertility, environmental factors like stress at work, smoking, pelvic surgery, chronic medical disorder, indication of IVF, basal FSH, mean age of menopause in their mothers etc.

**RESULTS:**

Among the poor responders, 60.57% were above 35 years of age compared to 36.41% in control group, which is statistically significant. Mean age of menopause in mother was found to be four years earlier in poor responder group. Male factor and unexplained infertility were significantly (*P*<0.05) higher in good responders (*P*<0.05). Significant proportion (31.73%) of women in study group had undergone some pelvic surgery (*P*<0.05).

**CONCLUSION:**

Apart from age, prior pelvic surgery also could be used as predictors for poor ovarian response. Heredity also plays a major role in determining ovarian response.

## INTRODUCTION

Ovarian response can be defined as the reaction of the ovaries to exogenous stimulus. It varies considerably among individuals and in the same individual as well.[1] Ovulation induction and controlled ovarian stimulation aim at achieving a distinct ovarian response, which predicts the success of such treatment.[2] To select best embryos, it is important to obtain several embryos[3,4] Poor response in IVF yields lesser oocytes and is associated with poorer pregnancy perspective. Older patients and patients with elevated basal FSH are known to respond poorly owing to the poor ovarian reserve.[5] Younger patients with no obvious cause are also found to respond poorly in IVF. In fact, poor responders can be those with low ovarian reserve and those with normal ovarian reserve but inherent low response to gonadotrophin.[1] Although no definite cause could be attributed, differences in metabolism of exogenous FSH could be responsible for the varying ovarian response.[6] Hence, age cannot be taken as a sole predictor of cumulative pregnancy to identify poor prognosis cases.[7] There is a need for predictors of poor response which could be used to reduce cycle cancellation rate. Antral follicle count may be considered as the best single predictor for poor ovarian response than age and endocrine markers.[8] Apart from basal FSH and AFC, several other factors like basal inhibin and AMH are currently being investigated as predictors of ovarian response. There are various female and male lifestyle habits, which could possibly affect success rates in ART. Smoking has been proved to affect IVF outcome negatively whereas role of stress, caffeine and alcohol is inconclusive[9]

Cycle cancellation due to poor response is frustrating for both clinician and the patient and demands meticulous follow up. Studies have shown that women conceiving after poor ovarian response have more pregnancy complications like PIH and preeclampsia than women with normal ovarian response.[10] In addition, poor ovarian response could be a predictor of early menopause.[11] This paper studies various demographic and clinical profiles of poor responders and tries to look at the known and unknown factors, which could contribute to poor ovarian response in IVF.

## MATERIALS AND METHODS

Patients undergoing IVF/ICSI from Jan 2008 to Jan 2009 were included in the study. Patients having lesser than four oocytes at retrieval and those cycles which were cancelled due to poor follicular development, were defined as poor responders.[12] 104 poor responders according to the above criteria were included in the study and were compared with 324 good responders. These patients had been allocated to different protocols depending on the basal FSH, AFC and age (Antagonist / GnRHa long protocol). The protocol for pituitary down regulation, ovulation induction, and HCG administration was consistent throughout for both the study and the control group.

Data were collected retrospectively from the records of all 104 poor responders and compared with those of the good responders. We looked at various demographic characteristics like age, BMI, type of subfertility, duration of subfertility, environmental factors like stress at work and smoking in any of the partners. Both groups were also compared for clinical profile like pelvic surgery, chronic medical disorder, and indication of IVF, basal FSH, and mean age of menopause in their mother.

### Study design

Retrospective comparative study.

### Statistical analysis

GraphPad QuickCalc was used for the data computation. Chi-square test and Fisher exact test were used for comparing categorical data. *P* value of less than 0.05 was taken to be significant.

Unpaired *t* test was used to compare continuous data.

## RESULTS

Among the poor responders, 60.57% were above 35 years of age compared to 36.41% in control group, which is statistically significant. Incidence of primary subfertility in the two groups was similar [Table 1]. More than half of women in poor responders group were in stressful jobs as compared to the control group. Most of the women came from the urban set up in both groups but significantly higher in study group (*P*=0.01). Smoking in either of partner could not be specifically correlated to any of the groups (*P*=0.10).

Mean age of menopause in mother was found to be four years earlier in poor responder group, which is statistically significant [Table 1]. Poor responders were found to have prolonged subfertility compared to the control group (*P*<0.05).

**Table 1:** Demographic characteristics

Factors	Poor responder Good responder *n*=104	Good responder Good responder *n*=324	*P* value
Age >35 years	63 (60.57)	118 (36.41)	0.01
Primary subfertility	93 (89.42)	247 (76.23)	0.37
Working women	57 (54.8)	116 (35.8)	0.03
Urban	90 (86.53)	183 (56.48)	0.01
Smoking (in any partner)	19 (18.26)	95 (29.32)	0.1
Mean age of menopause in mother	38±1.23	42±2.35	< 0.05
Mean duration of subfertility	11±4.32	7±2.86	< 0.05
BMI	26±1.39	23±2.9	0.06

Figures in parentheses are in percentage

### Causes of infertility

Major indication of IVF in poor responder group was multifactor [Table 2] and hence the other causes as sole factor were less found among them. Male factor and unexplained infertility were significantly (*P*<0.05) higher in good responders (*P*<0.05). In contrast, unexplained and PCOS were rare among poor responders. Incidence of endometriosis was similar in both groups; 25.96% women in poor responder group had high basal FSH (>10iu) which is statistically extremely significant. Majority of women in both groups had regular periods and had good endometrial development [Table 3].

**Table 2:** Causes of infertility

Factors	Poor responder *n*=104	Good responder *n*=324	*P* value
Indication of IVF			
Male factor	11 (10.57)	107 (33.02)	< 0.05
Tubal factor	15 (14.42)	84 (25.92)	0.06
Endometriosis	10 (09.15)	9 (2.96)	0.15
Multifactorial	65 (62.5)	50 (15.43)	< 0.05
Unexplained	3 (2.88)	54 (15.74)	< 0.05
PCOS	0	36 (11.11)	< 0.05

Figures in parentheses are in percentage

**Table 3:** Clinical profile

Factors	Poor responder *n*=104	Good responder *n*=324	*P* value
FSH > 10iu	27 (25.96)	11 (3.39)	< 0.05
Endometrial thickness >8mm	95 (91.34)	292 (90.12)	0.99
Regular cycles	98 (94.23)	235 (72.53)	0.13

Figures in parentheses are in percentage

### Medical or surgical illness

Significant proportion (31.73%) of women in study group had undergone some pelvic surgery (*P*=0.01). Prevalence of diabetes and hypertension were similar between two groups. Among different types of surgeries, excision of endometriotic cyst was the major surgery in poor responder as opposed to ovarian drilling for PCOS in good responders [Table 4].

**Table 4:** Medical or surgical illness

Factors	Poor responder *n*=104	Good responder *n*=324	*P* value
H/o. Pelvic surgery	33 (31.73)	56 (17.28)	0.01
Chronic medical disorders			
Diabetes	7 (6.73)	29 (8.95)	0.65
Hypertension	3(2.88)	10 (3.08)	0.91

Figures in parentheses are in percentage

## DISCUSSION

Stress and infertility are closely associated. Stress in life may predispose to infertility, whereas stress of infertility affects quality of life in both partners equally.[13] Working women are exposed to stress of travelling, environmental pollution and passive smoking in addition to stress at work. Various negative life events preceding IVF can affect adversely the outcome including number of oocytes harvested during retrieval.[14] Environmental pollution can affect the sperm parameters adversely thereby leading to infertility.[15] As evident from our study, poor responders group has majority of working women and most of them come from an urban set up. Effect of smoking is inconclusive from our study as most of the women who were included in this category had passive smoking only as smoking among middle class Indian women is rare. Prolonged sub fertility among poor responders specifies that in addition to age, there is some other unknown but significant factor, which plays a role. This decreased response could be attributed to a hereditary factor as the mean age of menopause in their mothers is significantly lower than those in the good responders. Studies have shown that expression of follicle stimulating hormone receptor (FSHR) in granulosa cells varies among poor, moderate and high responders. Low expression of FSHR at the mRNA level in poor responders plays a critical role and can be correlated to the role of heredity as seen in our study.[16–18]

Factors causing subfertility perhaps do not affect outcome of ovarian response except endometriosis. In fact, endometriosis is a major cause of poor response in IVF in younger patients. But in our study both the groups have comparable number of patients with endometriosis. This may be due to the fact that most of the poor responders have multiple factors causing infertility, which includes endometriosis as well. In contrast, patients with unexplained subfertility do not present with poor response usually [Table 3] as the defect in these patient may be ovulatory, peritoneal, fertilization or implantation related. Although significant number of patients in study group had higher basal FSH, their endometrial development is comparable with their counter parts, indicating that they represent a group of normally ovulating women.[19,20]

Effect of chronic medical disorders like diabetes and hypertension is very little on the ovarian response as seen in our study. Prior pelvic surgery poses a major risk factor for poor ovarian response as there is a significant correlation of pelvic surgery and poor ovarian response (*P*=0.01) in our study. This may be due to insult to the ovarian vascularity during such surgeries. Vascularity could be compromised by direct injury during surgery or due to post-operative infection leading to micro thromboembolism.

The major drawback of this study is probably the different protocols used for different groups of patients. Till now, there is no clear consensus about an ideal stimulation regimen for poor responders, as retrospective analysis of various protocols used for them did not reveal any differences.[21] Some studies have indicated superiorly of antagonist protocol in terms of reduced duration of ovarian stimulation, less use of GTH, fewer cycle cancellation and more oocyte yield,[22] whereas others have shown the advantages of using short agonist protocol. In our practice, we have observed fewer cycle cancellation with antagonist protocol and hence used antagonist protocol for the patients who were having low AFC and high FSH and were expected to have poor response. Studies have proved that increasing the dose of gonadotrophin does not rectify poor response; hence, amount of gonadotrophin used has not been taken into account in our study.[5,23]

## CONCLUSION

Poor responders are a challenge to the IVF specialists and need to be investigated for the cause. Age is an established indicator of poor response as with advancing age there is depletion of primary oocytes. Apart from age, endometriosis and prior pelvic surgery also could be used as predictors for poor ovarian response. Above all, heredity also plays a major role and needs to be investigated further.
